# Bacillus subtilis spore vaccines displaying protective antigen induce functional antibodies and protective potency

**DOI:** 10.1186/s12917-020-02468-3

**Published:** 2020-07-28

**Authors:** Yeonsu Oh, Jung Ae Kim, Chang-Hwan Kim, Soo-Keun Choi, Jae-Gu Pan

**Affiliations:** 1 Department of Veterinary Pathology, College of Veterinary Medicine and Institute of Veterinary Science, Kangwon National University, 1 Kangwondaehak-gil, Chuncheon, 24341 Republic of Korea; 2 Infectious Disease Research Center (Superbacteria Group), Korea Research Institute of Bioscience and Biotechnology (KRIBB), 125 Gwahak-ro, Yuseong-gu, Daejeon, 34141 Republic of Korea; 3 The 4th R&D Institute, Agency for Defense Development (ADD), Yuseong, Daejeon, 34186 Republic of Korea

**Keywords:** Native display, *Bacillus*, Spore, Protective antigen, Anthrax, Mucosal vaccine

## Abstract

**Background:**

*Bacillus anthracis* is the causative agent of anthrax, a disease of both humans and various animal species, and can be used as a bioterror agent. Effective vaccines are available, but those could benefit from improvements, including increasing the immunity duration, reducing the shot frequency and adverse reactions. In addition, more sophisticated antigen delivery and potentiation systems are urgently required.

The protective antigen (PA), one of three major virulence factors associated with anthrax was displayed on the surface of *Bacillus subtilis* spores, which is a vaccine production host and delivery vector with several advantages such as a low production cost, straightforward administration as it is safe for human consumption and the particulate adjuvanticity. Mice were immunized orally (PO), intranasally (IN), sublingually (SL) or intraperitoneally (IP) with the PA displaying probiotic spore vaccine. Clinical observation, serological analysis and challenge experiment were conducted to investigate the safety and efficacy of the vaccine.

**Results:**

A/J mice immunized with the PA spore vaccine via PO, IN, SL, and IP were observed to have increased levels of active antibody titer, isotype profiles and toxin neutralizing antibody in sera, and IgA in saliva. The immunized mice were demonstrated to raise protective immunity against the challenge with lethal *B. anthracis* spores.

**Conclusions:**

In this study, we developed a *B. subtilis* spore vaccine that displays the PA on its surface and showed that the PA-displaying spore vaccine was able to confer active immunity to a murine model based on the results of antibody isotype titration, mucosal antibody identification, and a lethal challenge experiment.

## Background

*Bacillus anthracis* is a nonmotile, facultative anaerobe that occasionally infects humans, rather anthrax is most often a veterinary concern especially among field-grazed herbivores [1] and the live vaccine for anthrax has widely been used in the veterinary arena with various herbivore species. Comprehensive understanding and development of vaccines for humans were investigated vigorously by various countries, and the potential vicious use of *B. anthracis* as a biowarfare agent prompted to push towards better anthrax vaccines for humans, not livestock [2].

The currently available anthrax vaccines are alum-precipitated *B. anthracis* Sterne strain crude culture filtrates (AVP; Anthrax Vaccine Precipitated) or the AVA (Anthrax Vaccine Adsorbed) adsorbed onto aluminium hydroxide consisting mainly of protective antigen (PA) from cultures of the unencapsulated, toxin-producing *B. anthracis* V770-NP1-R strain [3]. Both vaccines require multiple injections intramuscularly and a yearly boost, and leave local reactogenicity at injection site. In addition, more sophisticated antigen delivery and potentiation systems are urgently necessary [4]. Although the anthrax vaccine is not currently recommended for the general population, future incidents and applying for veterinary use could result in the re-evaluation of the vaccine and its recommendations.

PA is a critical, cell-binding component for the transport of edema and lethal toxins into a targeted cell, of which domains 1b and 4 are known to contain protective epitopes, and immunization with recombinant PA has been shown to induce protection against *B. anthracis* infection [2, 5].

*Bacillus subtilis* spores have successfully been used as a probiotic for both humans and animals [6] and have been employed as vectors for the mucosal delivery of vaccine antigens [7]. Spore vaccines offer a myriad of advantages such as aid in mass vaccinations by increasing ease and speed of delivery, decreased costs by removing purification steps, flexible administration via mucosal or oral routes, thus providing ‘needle-free’ and ‘refrigeration-free’ vaccine delivery systems [8, 9]. Another unique feature is that spores have sub-micron scale nanostructures, allowing them to serve as effective particulate adjuvants [10]. Particulate adjuvants, such as liposomes, virosomes, virus-like particles, poly-lactide-co-glycolide (PLG) microspheres and immune stimulating complexes (ISCOMS), sufficiently target antigen presenting cells (APCs) and once internalized within the cell are processed by the class I and class II MHC (major histocompatibility complex) pathway leading to antigen presentation on the surface of the APC [11]. Studies to investigate the adjuvanticity of *B. subtilis* spores proved that strong auxiliary effects were observed when co-administered with protein antigens either admixed or adsorbed on the spore coat surface [10]. Other studies have demonstrated that orally administered *B. subtilis* spores germinate in the murine gut, disseminate to the gut-associated lymphoid tissue (GALT), and enter Peyer’s Patches and mesenteric lymphoid tissues [12, 13]. Spores displaying antigens were also evidenced to confer a germination independent immune response [14]. The resilience of spores, coupled with a mucosal route of delivery, make spore vaccines promising candidates for emergency use in developing countries and in response to bioterrorism.

Here, we present a novel probiotic spore vaccine that displays PA in its native form on the *B. subtilis* spore surface; the design of the vaccine represents an innovative concept for native protein display that does not require the generation of a fusion protein [15]. In this system, target PAs are highly expressed in the mother cell compartment at the sporulation phase and are attached or adsorbed to the spore surface, similar to normal coat proteins [16]. We investigated the safety and efficacy of the PA-displaying spore vaccine administered per orally (PO), intranasally (IN), sublingually (SL), and intraperitoneally (IP), and the route of challenge was taken through subcutaneous route to produce more serious infection. As the route of vaccine administration has a significant effect on the nature of the host immune response, we researched the systemic and mucosal immunogenicity and resistance to lethal challenge with anthrax spore depending on inoculation routes.

## Results

### PA displayed on the spore surface

**Fig. 1:**
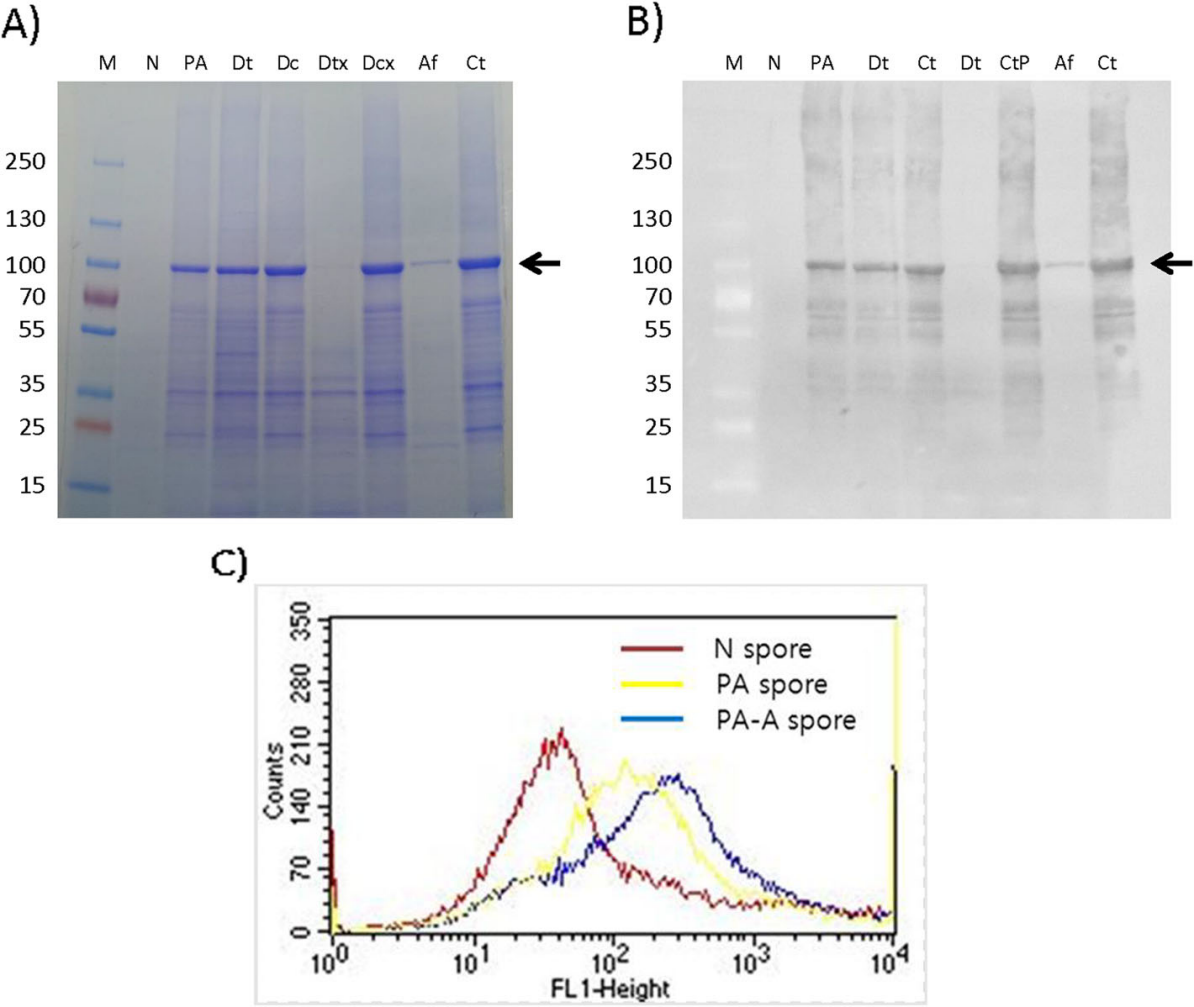
Expression and display of PAs. **a** SDS-PAGE and **b** Western blot images of PAs detached and decoated from spore surface. **c** Flow cytometry histogram for N spore (control spore), PA spore (PA-displaying spore), and PA-A spore (PA naturally displayed and adsorbed again on the spore). Abbreviation: M, Marker; N, N spore, *B. subtilis* spore; PA, Protective antigen displaying *B. subtilis* spore; Dt, NaCl treated detached protein; Dc, NaCl treated coat protein; Dtx, Triton X-100 treated detached protein; Dcx, Triton X-100 treated coat protein; Af, after adsorption; Ct, coat protein. Arrow, size of the PA protein

Some of the PA was detached from spore surface when treated with the high concentration of NaCl solution, but most of the PA remained on the spore surface when treated with the detergent (Triton X-100 solution) added NaCl solution (Fig. 1a). Compared to the control N spore, flow cytometry showed more PA specific signal on the PA expressing spore, and adsorbing additional PA (PA-A) increased the signal further confirming the specificity to PA. The histogram of the PA spore shifted toward the right and the PA-A spore was more skewed to the right, indicating that this shift is due to displayed PA on the spore surface (Fig. 1b).

### Serum antibody response by administration route

**Fig. 2:**
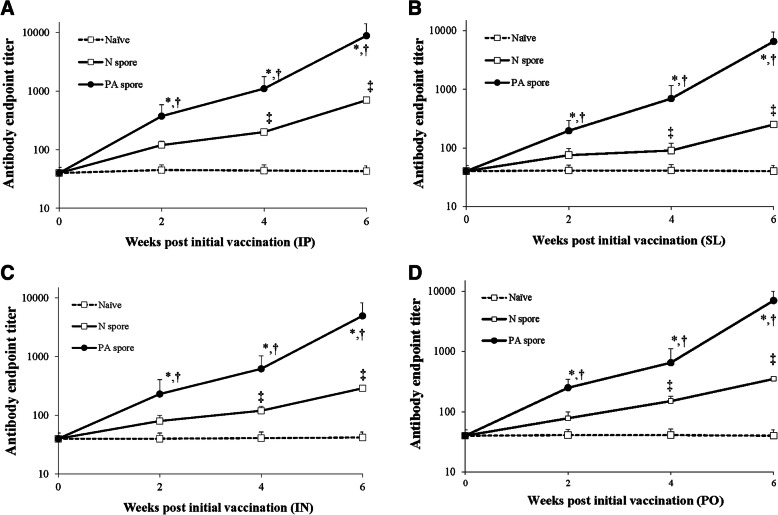
Serum antibody responses for groups receiving different administration routes; **a** IP route, **b** SL route, **c** IN route, and **d** PO route. Mice were immunized with the PA antigen displaying spore vaccine, and the serological responses were monitored. *, significant (*P* < 0.05) difference at each time point versus the previous time point within a group. †,‡, significant (*P* < 0.05) difference between groups at the same time point. Mean ± standard error. Abbreviation: Naïve, PBS treated group; N spore group, non-recombinant *Bacillus subtilis* spore treated group; PA spore group, PA antigen displayed *Bacillus subtilis* spore treated group

The antibody endpoint titers were significantly increased at each time point in the PA spore groups, regardless of the administration route (IP, Fig. 2a); SL, Fig. 2b); IN, Fig. 2c); PO, Fig. 2d)). At 6 weeks post initial vaccination, the PA spore vaccine treated groups for all inoculation routes showed increased serum immunogenicity against the PA antigen compared with the Naïve and N spore groups (*P* < 0.05). The PA spore groups for all administration routes presented significantly increased antibody titers compared with the pre-inoculation titers and compared with the antibody titers of the other groups at the same time points. However, the antibody titers of the N spore groups were presented higher than those of the Naïve group, when administered via IP, IN, and PO (*P* < 0.05).

### Anti-PA antibody isotype profiles in serum

**Fig. 3:**
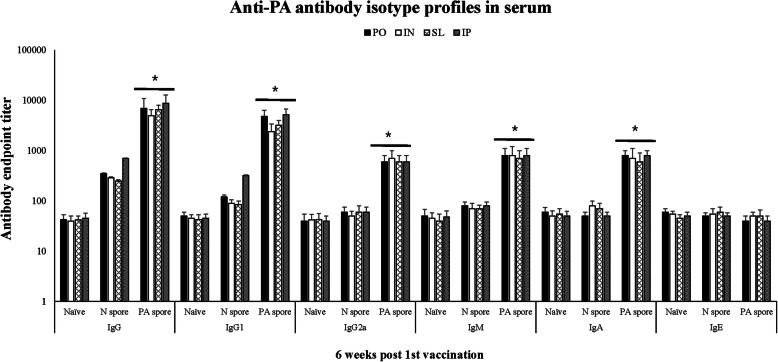
Anti-PA antibody isotype profiles in serum from animals treated with different vaccine administration routes at 6 weeks post vaccination (6wpv). The serological responses were monitored, and the results at 6 wpv are presented. *, †, significant (*P* < 0.05 and *P* < 0.01) difference between groups at the same time point. Mean ± standard error. Abbreviation: Naïve, PBS treated group; N spore group, non-recombinant *Bacillus subtilis* spore treated group; PA spore group, PA antigen displayed *Bacillus subtilis* spore treated group

The anti-PA IgG, IgG1, IgG2a, IgM, and IgA concentrations except IgE in the PA spore-treated groups were significantly higher than in the N spore and Naïve groups regardless of the route of administration (*P* < 0.05) as shown in Fig. 3. The IgE levels in all routes of administration did not differ among groups.

### Mucosal antibody responses

**Fig. 4:**
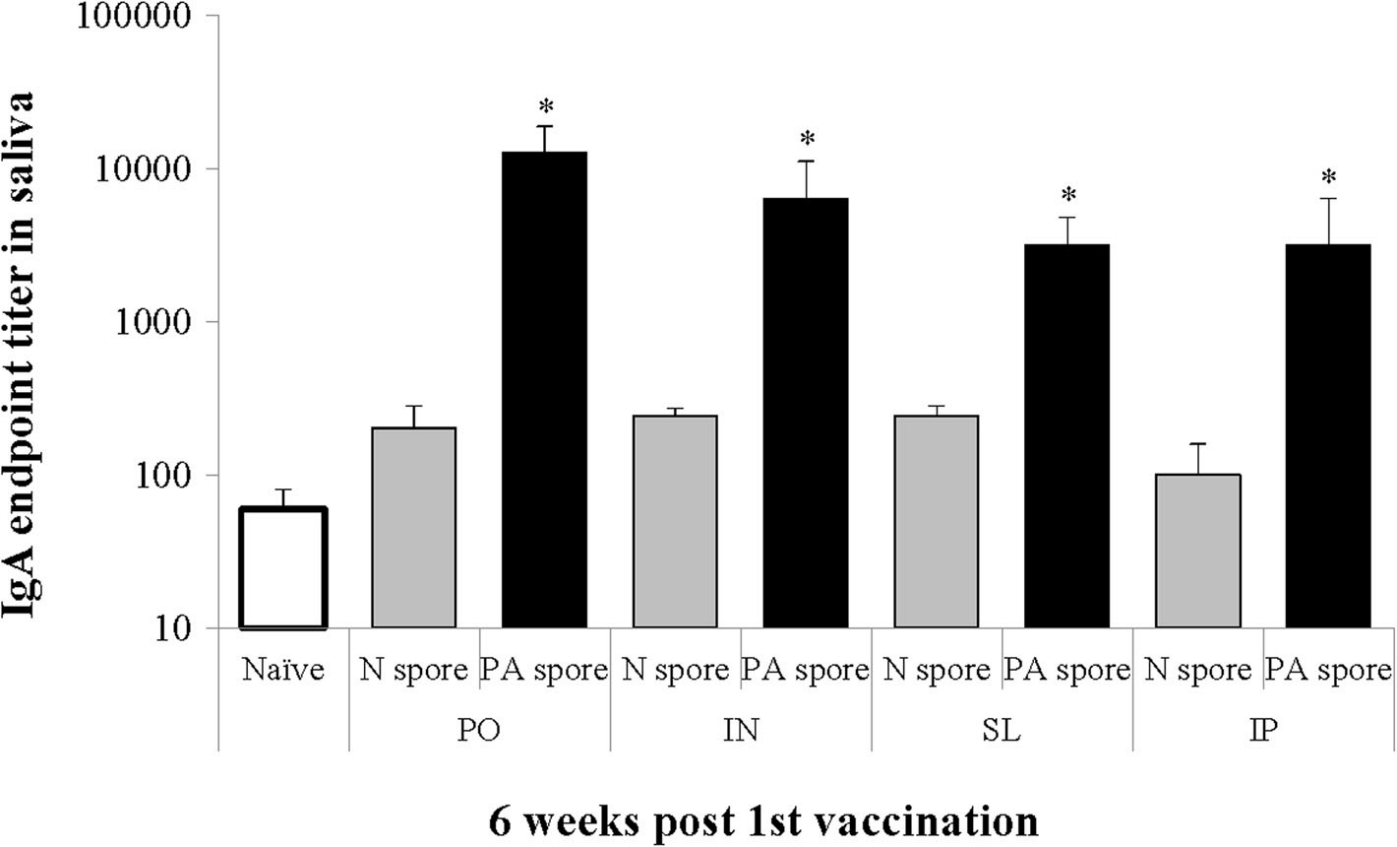
Anti-PA IgA response in saliva. *, significant (*P* < 0.05) difference between groups at the same time point. Mean ± standard error. Abbreviation: Naïve, PBS treated group; N spore group, non-recombinant *Bacillus subtilis* spore treated group; PA spore group, PA antigen displayed *Bacillus subtilis* spore treated group

The anti-PA IgA concentrations in the saliva were significantly increased in the PA spore group administered via PO, IN, SL, and IP (*P* < 0.05; Fig. 4). No significant differences were observed between administration routes in the PA spore group.

### Toxin neutralizing antibody (TNA) responses

**Fig. 5:**
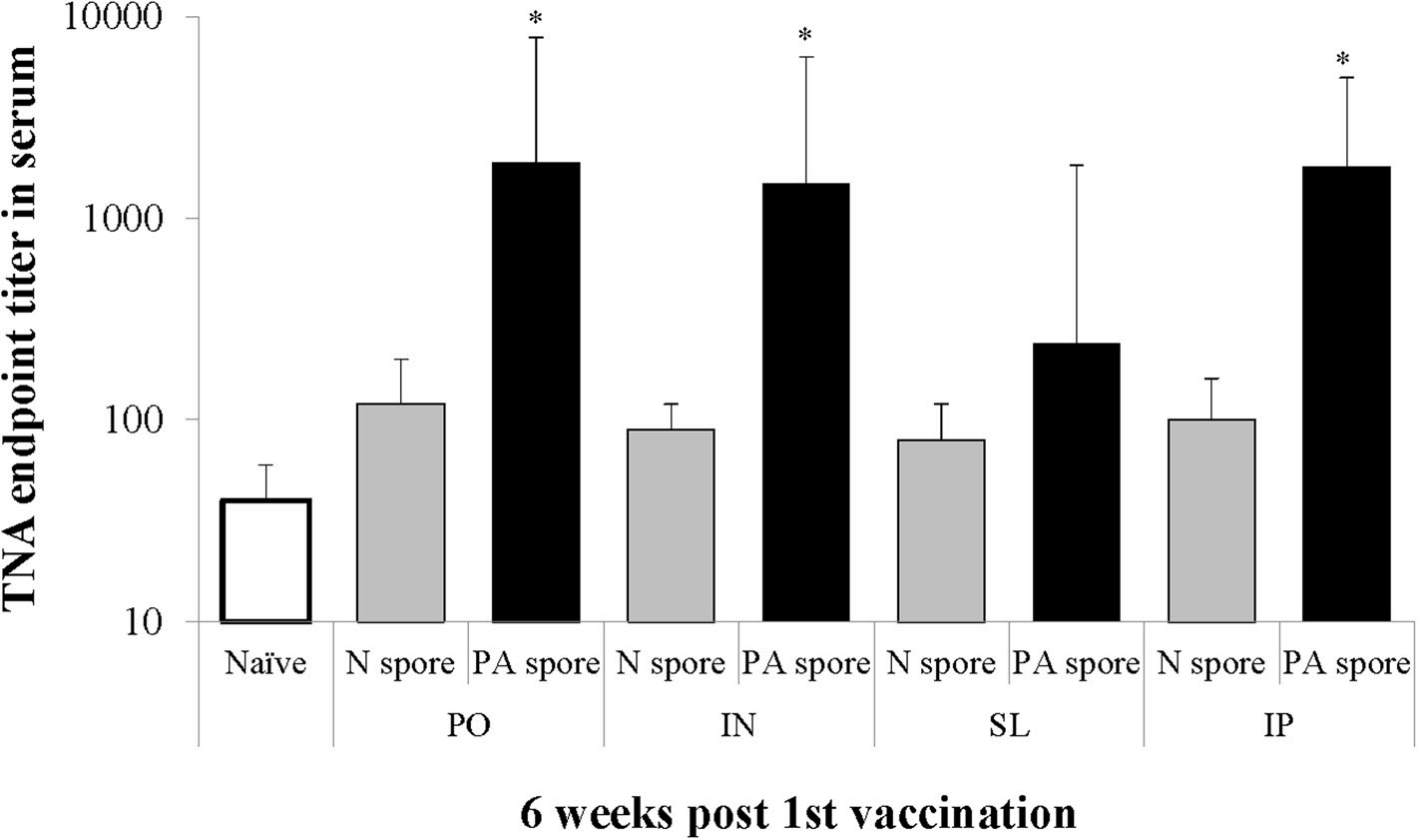
Toxin neutralizing antibody responses in serum. *, significant (*P* < 0.05) difference between groups at the same time point. Mean ± standard error. Abbreviation: Naïve, PBS treated group; N spore group, non-recombinant *Bacillus subtilis* spore treated group; PA spore group, PA antigen displayed *Bacillus subtilis* spore treated group

The TNA in the PA spore group inoculated via PO, IN, and IP were significantly higher than those of the Naïve and N spore groups (*P* < 0.05; Fig. 5). No significant differences were observed between three routes of inoculation except SL in the PA spore group. The SL group had lower response than the other routes. In addition, the N spore groups were all higher than the Naïve as seen in the IgG response.

### Survival rate after challenge with lethal *B.anthracis* spores

**Fig. 6:**
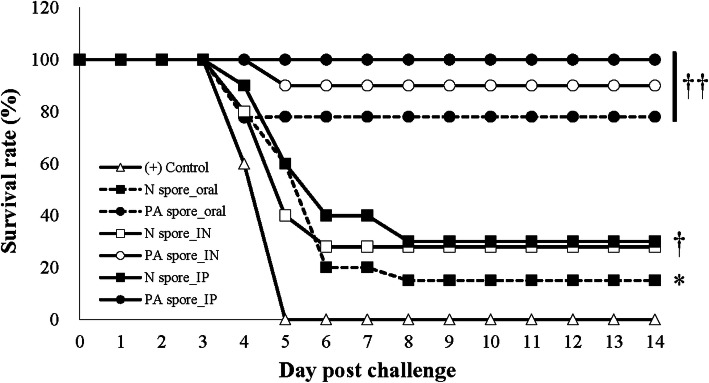
Survival of mice immunized with the PA spore displaying vaccine and challenged with 6 × 10^7^ CFU (Tox + Cap−) spores of the *B. anthracis* Sterne strain (equivalent to 100 50% median lethal doses (MLD_50_s) per animal. ††, *P* < 0.01; †, *P* < 0.05; *, *P* < 0.05

Mice immunized with PA spore vaccines were shown to be protected significantly against the challenge regardless of the route of administration compared with the N spore and Naïve groups (*P* < 0.01). The N spore groups were shown to be partially protected significantly compared with the Naïve group (*p* < 0.05) and the N-IP and N-IN groups were significantly protected compared with the N-PO group by inter-group comparison (*P* < 0.05). The naive mice presented anthrax symptoms and died within 2–5 days after the challenge (Fig. 6). The relative protective level of the different routes was the same in both the PA and N spore groups (IP highest, followed by IN and oral), suggesting a real effect of route.

## Discussion

The anthrax vaccine is recommended for military personnel, lab personnel, environmental workers, and handlers of animals or animal products who experience higher risks of exposure to anthrax spores (https://www.cdc.gov/vaccines/vpd/anthrax/index.html). Documented reactions include nearly 50 different types of major side effects (http://www.publichealth.va.gov/exposures/gulfwar/vaccinations.asp). Anthrax vaccines could be improved by supplementing the following: to confer better local immune responses against target pathogens to invade the body primarily through mucosal barriers, to provide needle-free routes of administration, to offer improved safety with minimal adverse effects, and to provide economical vaccines for developing countries, not requiring refrigeration during storage and transportation resulting in effective immunization programs [17].

Protein display is a genuine technique to present target proteins on the surface of microorganisms [18]. Since the first successful display of an antibody and its library on a phage surface, protein display technology has been applied in a wide variety of applications, including peptide and/or antibody library screening, vaccine development, and mass production of biological products such as enzymes [19]. For efficient display, target proteins should be fused to display motifs and be translocated across the cell membrane before being anchored to the membrane surface [20]. Display of the PA on *B. subtilis* spores was enabled by fusing proteins with spore surface coat proteins, such as CotB, CotC, CotG, or CotZ [21]. In the study, the highly expressed target PA was displayed on the surface of *B. subtilis* spores in its native form without fusing to avoid the risk of failing to obtain a functional construct.

A probiotic grade of *B. subtilis* spores could be utilized as a mucosal vaccine delivery system and simultaneously as an adjuvant for mucosal immunity for the following reasons: 1) *B. subtilis* spores are resistant at ambient temperatures without risking the loss of viability; 2) *B. subtilis* spores are safe enough for consumption by humans as food components, probiotics, or therapeutics; 3) *B. subtilis* can be genetically manipulated, making it possible to engineer bacteria that express and display immunogens on the spore surface or in the vegetative cells; and 4) *B. subtilis* spores can serve as a non-invasive vaccine delivery systems [2, 10, 21]. Such spore vaccine adjuvants would be particularly useful in regions where cold-chain transportation is difficult or emergency biothreat situations. Probiotic *B. subtilis* spores have been mentioned to be immunostimulatory in many other studies [10, 21–23]. Interestingly, when given at a high dose, spores alone were able to protect against an H5N1 virus challenge in a mouse model [23, 24]. Those results were consistent with the results in our study. Mice in the PA spore group as well as N spore group presented meaningful antibody isotype profiles in sera. Particularly notable was the anti-PA specific IgA response observed in the saliva. The PA spore group implemented increased IgA in saliva compared with the N spore and Naïve groups via all routes tested. The results are consistent with other studies that needle free vaccines can induce immune responses at both systemic and mucosa levels [25]. IgA is more broadly protective than other immune molecules against foreign invaders entering our bodies through mucosal barriers [26].

In general, vaccines delivered via mucosal routes are poorly immunogenic because these antigens are easily degraded by mucosal or intestinal enzymes [27]. However, considering the route of infection, the development of a mucosal adjuvant and/or a delivery vehicle for mucosal immunity seems to be critical to manufacture a vaccine against anthrax. PO, IN and SL immunization has been shown to induce strong systemic and secretory antibody responses, and particularly, IN and SL routes require considerably smaller doses of antigen than would an oral administration [24, 28]. Orally administered recombinant lactococci have been used successfully to elicit systemic antibody responses against tetanus toxin fragment C and the induced IgG subclasses IgG1 and IgG2a pointed to the importance of determining the types of antigen-specific T-helper subset responses [29]. The resulting anti-PA antibodies in our study successfully reached protective levels against a lethal challenge with *B. anthracis*, indicating that the PA displaying *B. subtilis* spore vaccine be capable of eliciting functional immunity via mucosal routes. In addition, it means the spore vaccine raise the cellular immunity as well, i.e., specialized functional B cells. However, the SL route induced lower TNA response, although the SL route induced favorable antibody responses. It suggests that the route somehow changes the epitope selection to non-neutralizing epitoes. It remains to be explored more in the future.

*B. subtilis* spore has been an attempt as a vaccine adjuvant by other research groups, but our study compared the effects of variable mucosal administration routes and the challenge was attempted subcutaneously to induce more powerful infection rather than an inhalation route. As a result, spores displaying PA antigen provided full protection to experimental animals when administered via various mucosal routes, and wild type spore (N spore) also conveyed partial immunity to animals.

## Conclusion

In conclusion, the established PA displaying *B. subtilis* spore vaccines represent an easy to produce, practical to handle, human-safe and an economically feasible opportunity to provide protection from anthrax to human and animal populations. For further study, it would be interesting to explore if there are any particular spore types or *Bacillus* strains showing enhanced immunity with antigens displayed on the spore surface. Further discussion remains to be dedicated to other promising *B. anthracis* antigens and immunization routes that may lead to longer-lasting, more-efficacious vaccines with available technology.

## Methods

### Plasmids, strains, and culture conditions

A modified *cry3Aa* promoter (P_5D_), containing a consensus sequence in the − 35 region, was used to express and display the target protein on the surface of *Bacillus* spores during the sporulation phase [16]. The rPA gene was synthesized by a commercial vender (Bioneer, Seoul, Republic of Korea). All DNA manipulations were performed in *Escherichia coli* JM109 competent cells (Takara bio inc., Tokyo, Japan). The *B. subtilis* strains DB104 [30] and WB800N [31] purchased from MoBiTec (Goettingen, Germany) were used as host strains. *B. subtilis* sporulation was achieved by incubation in Difco sporulation medium (DSM)(Difco, Becton, Dickinson and Company, NJ, USA)(8 g nutrient broth, 0.1% KCl, 0.012% MgSO_4_, and 1% NaOH in 1 L of distilled water, supplemented with 1 mM Ca(NO_3_)_2_, 10 μM MnCl_2_ and 1 μM FeSO_4_•7H_2_O) for 24–36 h at 37 °C. Ampicillin (100 μg/ml) or chloramphenicol (5 μg/ml) was added to the medium when required.

### Construction of PA producing *B. subtilis*

PA producing *B. subtilis* was constructed as previously described [32]. Using the plasmid pMar3g as a template, the promoter P_5D_ and the *pagA* gene were fused and ligated to construct the plasmid pD5D-*pagA*. The pD5D-*pagA* plasmid was finally introduced into *B. subtilis* WB800N, inserting the P_5D_-*pagA* expression cassette into the *amyE* locus.

### Spore preparation and verification of PA on the spore surface

The expression of PA and the sporulation of PA-producing *B. subtilis* were monitored during batch cultivation in DSM at 30 °C by measuring the number of spores using a hemocytometer and a microscope. A greater than 70% of sporulation efficiency was observed, spores were purified, as previously described [9]. Briefly, after sufficient sporulation was observed, vegetative cells were lysed and spores were harvested by centrifugation at 10,000 rpm for 10 min, followed by washing twice with phosphate-buffered saline (PBS, pH 7.0). Then, washed spores were treated with lysozyme (50 μg/ml) for 30 min at 37 °C to analyze the presence of PA in spore coat protein by a Western blot assay, using a polyclonal anti-PA antibody (Cat.# LS-C19484, WA, US). Also, NaCl (1 M) and Triton X-100 (0.1% in NaCl, 1 M) were treated on the PA spore and sonicated for decoating purpose. Then, the detached and remained PA was measured by the Western blot assay.

The surface display of PA was also analyzed through flow cytometry. For immunofluorescence staining, the purified spores were washed three times with PBS, resuspended in 1 ml of PBS containing 5% (w/v) skim milk, and incubated for 1 h at 37 °C to block nonspecific antibody binding. After being washed with PBS, the spores were incubated for 1 h at 37 °C with anti-PA mouse monoclonal antibody (Cat.# MBS190056, MyBioSource.com, CA, US) specifically targeting the PA protein, in PBS supplemented with 0.5% (w/v) skim milk. Then, the spores were washed with PBS and incubated with fluorescein isothiocyanate (FITC)-conjugated anti-mouse IgG (1:200, Sigma-Aldrich, MO, USA) for 1 h at 37 °C. The samples were washed three times, resuspended in 1 ml of PBS, and analyzed using a FACSort flow cytometer (Becton Dickinson, CA, USA) and CellQuest ver.1.0 software. In the same way, to amplify the bias effect to the right on the FACS analysis of the PA, PA-displayed spore was adsorbed with concentrated crude PA protein (PA-A spore) and measured by FACS.

### Animals

Specific-pathogen-free, age-matched male A/J mice (22 ~ 25 g in weight; 8 ~ 10 weeks in age) were obtained from Orient Bio (Sungnam, Republic of Korea) and Charles River Laboratories (Hollister, CA, USA), respectively and maintained at a constant temperature (21 ± 2 °C) and a 12–12 h light–dark schedule under specific pathogenic-free conditions in the animal facilities of the Korean Research Institute of Bioscience and Biotechnology (KRIBB; Daejeon, Republic of Korea). All animal experiments were performed blindly in accordance with the Institutional Guidelines for the Care and Use of Laboratory Animals in Research and the approval of the Animal Care and Use Committee of the KRIBB (KRIBB-AEC-13069).

Then, at various time points for each experimental purpose, mice were killed by administration of CO_2_.

### Immunizations

The prepared rPA spores were combined with Cholera toxin B (CTB; Sigma-Aldrich Co., MD, USA), which is a well-known as a mucosal delivery vehicle studied elsewhere and used in this study for the same purpose [33]. The sample size of experimental groups was calculated referring Sample Size Calculator Web application (https://www.surveysystem.com/sscalc.htm).

Groups of six mice were randomly selected to make groups according to weights and immunized either PO, IN, SL or IP with the recombinant PA-displaying spore vaccine. Each group was matched with a corresponding control groups that were treated with either a non-displaying spore vaccine (N spore) or with PA recombinant protein.

Oral doses of 5 × 10^9^ spores/100 μl of PBS were administered via intra-gastric lavage on days 0, 1, 2, 28, 29, 30, and 35, as adapted from the procedure described by Challacombe [24]. Nasal doses of 1 × 10^9^ spores/20 μl of PBS were dropped into the nostrils of mice using a micropipette, at 1–2 s intervals within a 30 s period, on days 0, 14, and 28. For SL doses, mice were lightly anesthetized by injection with Zoletil (100 mg/kg of body weight; Virbac, France) and xylazine hydrochloride (10 mg/kg of body weight; Bayer, Germany) and received a dose of 1 × 10^9^ spores/7 μl of PBS under the tongue on days 0, 14, and 28, as previously described elsewhere [34]. Briefly, forceps were placed under the tongue of the anesthetized mouse, and the vaccine was administered by a micropipette while the mouth was stretched open. After administration, mice were cautiously placed on their backs, to prevent swallowing until awakening from the anesthetic. IP doses of 1 × 10^9^ spores/100 μl of PBS were administered on days 0, 14, and 28. For all administration routes, control groups receiving non-recombinant spores (1 × 10^9^; N spore) and PBS (Naïve) were included.

### Sample collection

Serum and saliva samples were obtained at 0, 2, 4, and 6 weeks post vaccination (wpv). Blood was collected via the retro-orbital plexus after isoflurane induced anesthetization. The isolated serum samples were stored at − 20 °C until analysis. Saliva was collected as previously described [35]. Briefly, salivation was stimulated through the IP injection of 1 μg/g mouse pilocarpine hydrochloride (Sigma, MO, USA) in 100 μl of PBS. After 2 to 5 min, a micropipette was placed under the tongue to collect the salivary flow. The collected saliva was centrifuged at 10,000×g to remove any debris and stored at − 70 °C until analysis.

### Pretreatment to detect IgM and IgA

To improve the sensitivity and specificity of an indirect IgM and IgA ELISA, IgG was removed using recombinant protein G, according to the method described by elsewhere [26]. Briefly, 25 μl of serum was mixed with 100 μl of 50% Protein G coupled beads (50% [w/v] in PBS; Roche, Basel, Switzerland) and incubated for 1 h at 37 °C on a Nutator Mixer (BD Clay Adams, GA, USA). Then, the sample was centrifuged for 1 min at 1000×g and the 75 μl of supernatant, representing an approximately 1/3 dilution of the initial serum sample, was used for IgM and IgA analysis.

### Determination of anti-PA-specific antibody endpoint titration by the indirect ELISA

ELISA methods were used to measure antibody concentrations. First, 96-well microplates were coated with 50 μl of recombinant purified PA antigen (4 μg/ml in carbonate-bicarbonate buffer) per well and incubated at 4 °C overnight. After blocking with 2% BSA at room temperature for 1 h, serum samples were applied as a 2-fold dilution series, starting with a 1/40 dilution in assay dilution buffer (10 mM PBS [pH 7.4], 1% [w/v] BSA, 0.05% Tween 20). Every plate included replicate wells of a negative control (a 1/40 diluted preimmune serum).

The plate was incubated for 2 h at 37 °C, followed by washing with a washing buffer (10 mM PBS [pH 7.4], 0.05% Tween 20) for three times. Then, an anti-mouse IgG conjugated with FITC as a secondary antibody was diluted to 1:50 and distributed according to the manufacturer’s instruction. The plate was incubated for 1 h at 37 °C, followed by washing with a washing buffer (10 mM PBS [pH 7.4], 0.05% Tween 20) for three times. Absorbances were read on a spectrophotometry at 450 nm.

Dilution curves were extracted for each sample, and the endpoint titer for each sample was determined as the reciprocal of the dilution resulting in an optical density that was 0.1 U greater than that of the background value, as established by a 1/40 dilution of a pooled preimmune serum.

### Toxin neutralization assay (TNA)

The TNA was performed as described elsewhere with slight modifications [36]. Briefly, the same sera used in the antibody ELISA titration were measured using a RAW 264.7 cell line (ATCC® TIB-71™) instead of J774A.1. The RAW264.7 cell line was proved to be sensitive to the anthrax lethal toxin by others [12]. The anthrax lethal toxin (LeTx) consisted of 0.1 μg of PA and 0.08 μg of LF per ml in cell culture medium. The cell viability was measured using the MTT assay (Vybrant® MTT Cell Proliferation Assay Kit, ThermoFisher Scientific, MA, US) according to the manufacturer’s instruction. TNA titers were expressed as the reciprocal of the highest serum dilution which absorbance higher than the median absorbance of control wells (medium + LeTx)/2. Preliminarily, A 4-parameter sigmoid regression curve (Sigma Plot) was used to determine the dilution of the antisera that resulted in 50% neutralization (ED_50_) of anthrax LeTx. The neutralization ratio was determined by dividing the test sample ED_50_ by the reference sample ED_50_. A 4-parameter sigmoid regression curve was used to determine the dilution of anthrax LeTx that resulted in 50% cytotoxicity in the absence of serum from the toxin titration curve.

### Subcutaneous challenge with *B. anthracis* spores

Groups of 10 male A/J mice (8 ~ 12 weeks old) were challenged subcutaneously with approximately 6 × 10^7^ CFU (Tox + Cap−) spores of the *B. anthracis* Sterne strain (equivalent to 100 50% median lethal doses (MLD_50_s) per animal [13]. The animals were observed everyday for 14 days to determine their protected status. The challenge experiment was performed in a blinded fashion, and humane endpoints were strictly observed, such that any animal that displayed a collection of clinical signs that indicating a lethal infection was culled, and death was recorded. Individuals showing no symptoms after 14 days were considered immune.

### Statistical analysis

Statistical analysis was completed using IBM SPSS Statistics 24. Summary statistics were performed for all groups to assess the overall quality of the data, including normality. Obtained data were evaluated by a repeated measurement analysis of variance (ANOVA). If the test indicated significance, a one-way ANOVA with pair-wise testing using Tukey’s adjustment was performed for each time point. A value of *P <* 0.05 was considered significant.

## Data Availability

The data and material of this study is available from the corresponding authors on reasonable request.

## Abbreviations

PA: Protective Antigen
PO: Per orally
IN: Intranasally
SL: Sublingually
IP: Intraperitoneally
AVA: Aanthrax vaccine adsorbed
AVP: Aanthrax vaccine precipitated
PLG: Poly-lactide-co-glycolide
ISCOMS: Microspheres and immune stimulating complexes
APC: Antigen presenting cell
MHC: Major histocompatibility complex
GALT: Gut-associated lymphoid tissue
Cot: Coat proteins
MTT: Methyl thiazolyl diphenyl tetrazolium
